# Discontinued BACE1 Inhibitors in Phase II/III Clinical Trials and AM-6494 (Preclinical) Towards Alzheimer’s Disease Therapy: Repurposing Through Network Pharmacology and Molecular Docking Approach

**DOI:** 10.3390/ph19010138

**Published:** 2026-01-13

**Authors:** Samuel Chima Ugbaja, Hezekiel Matambo Kumalo, Nceba Gqaleni

**Affiliations:** 1 Traditional Medicine, School of Medicine, University of KwaZulu Natal, Durban 4000, South Africa; 2 Medical Biochemistry, School of Medicine, University of KwaZulu Natal, Durban 4000, South Africa; kumaloh@ukzn.ac.za; 3 African Health Research Institute (AHRI), 719 Umbilo Road, Durban 4001, South Africa

**Keywords:** Alzheimer’s disease, BACE1, drug repurposing, network pharmacology, molecular docking

## Abstract

**Background**: β-site amyloid precursor protein cleaving enzyme 1 (BACE1) inhibitors demonstrated amyloid-lowering efficacy but failed in phase II/III clinical trials due to adverse effects and limited disease-modifying outcomes. This study employed an integrated network pharmacology and molecular docking approach to quantitatively elucidate the multitarget mechanisms of 4 (phase II/III) discontinued BACE1 inhibitors (Verubecestat, Lanabecestat, Elenbecestat, and Umibecestat) and the preclinical compound AM-6494 in Alzheimer’s disease (AD). **Methods**: Drug-associated targets were intersected with AD-related genes to construct a protein–protein interaction (PPI) network, followed by topological analysis to identify hub proteins. Gene Ontology (GO) and KEGG pathway enrichment analyses were performed using statistically significant thresholds (*p* < 0.05, FDR-adjusted). Molecular docking was conducted using AutoDock Vina to quantify binding affinities and interaction modes between the selected compounds and the identified hub proteins. **Results**: Network analysis identified 10 hub proteins (CASP3, STAT3, BCL2, AKT1, MTOR, BCL2L1, HSP90AA1, HSP90AB1, TNF, and MDM2). GO enrichment highlighted key biological processes, including the negative regulation of autophagy, regulation of apoptotic signalling, protein folding, and inflammatory responses. KEGG pathway analysis revealed significant enrichment in the PI3K–AKT–MTOR signalling, apoptosis, and TNF signalling pathways. Molecular docking demonstrated strong multitarget binding, with binding affinities ranging from approximately −6.6 to −11.4 kcal/mol across the hub proteins. Umibecestat exhibited the strongest binding toward AKT1 (−11.4 kcal/mol), HSP90AB1 (−9.5 kcal/mol), STAT3 (−8.9 kcal/mol), HSP90AA1 (−8.5 kcal/mol), and MTOR (−8.3 kcal/mol), while Lanabecestat showed high affinity for AKT1 (−10.6 kcal/mol), HSP90AA1 (−9.9 kcal/mol), BCL2L1 (−9.2 kcal/mol), and CASP3 (−8.5 kcal/mol), respectively. These interactions were stabilized by conserved hydrogen bonding, hydrophobic contacts, and π–alkyl interactions within key regulatory domains of the target proteins, supporting their multitarget engagement beyond BACE1 inhibition. **Conclusions**: This study demonstrates that clinically failed BACE1 inhibitors engage multiple non-structural regulatory proteins that are central to AD pathogenesis, particularly those governing autophagy, apoptosis, proteostasis, and neuroinflammation. The identified ligand–hub protein complexes provide a mechanistic rationale for repurposing and optimization strategies targeting network-level dysregulation in Alzheimer’s disease, warranting further in silico refinement and experimental validation.

## 1. Introduction

Alzheimer’s disease (AD), as a progressively multifaceted neurodegenerative condition of the brain, is generally related to the impairment or death of neurons as the main pathophysiology. Memory impairment and erratic, illogical conduct are linked to another type of dementia, particularly in older adults over 60. Neurofibrillary tangles and β-amyloid plaques are the two hallmarks of AD. β-Amyloid precursor protein cleaving enzyme 1 (BACE1), also known as β-secretase, cleaves the amyloid precursor protein (APP) to produce β-amyloid plaques [1].

Since the discovery of BACE1 in 1999, it has become a major focus for developing drugs to inhibit or reduce β-amyloid aggregates in the brain. Reducing or inhibiting the accumulation of β-amyloid has long been the target in the design of drugs for AD treatment. Researchers had thought that designing strong, selective BACE1 inhibitors with minimal or no side effects might be made easier with a solid understanding of the distinctive features of BACE1 [1,2]. The Food and Drug Administration (FDA) has only approved five medications thus far to treat Alzheimer’s disease, and none of them target BACE1 [3]. Many previous and ongoing investigations have concentrated on BACE1’s therapeutic roles as a target in the management of AD during the roughly two decades since its discovery. Previous attempts have been made to create a few tiny pharmacological compounds that effectively inhibit BACE1. Verubecestat, lanabecestat, elenbecestat, and umibecestat (CNP-520) are a few of the BACE1 inhibitors that were first identified. Despite the fact that these inhibitors dramatically reduced β-amyloid plaques in patients with neurological Alzheimer’s throughout their phase 3 clinical trials, they were abruptly stopped due to health issues. The lack of BACE-targeted medications for the therapy of AD is partly due to the end of these clinical trials [4,5].

In 2019, a novel, potent, orally effective, and highly selective AM-6494 BACE1 inhibitor was discovered. This novel BACE1 inhibitor exhibited no fur coloration and common skin alteration, as observed with some initial BACE1 inhibitors. AM-6494, with an IC50 value of 0.4 nM in vivo, was selected and evaluated in preclinical phase trials [6]. Subsequently, the inhibition properties of this novel BACE1 inhibitor at the atomic and molecular levels of BACE1 were evaluated by ugbaja et al. [1,7]. Despite continued efforts to target BACE 1 in AD treatment, researchers have yet to design a successful BACE 1 inhibitor with appreciable efficiency and selectivity in the treatment of AD. AM-6494 was selected as a comparative benchmark due to its high potency and greater BACE1 selectivity over BACE2, resulting in improved side-effect profiles in preclinical models [6]. This contextualizes its role as a preclinical comparator to late-stage clinical failures (e.g., verubecestat, lanabecestat, elenbecestat, umibecestat), which failed not due to a lack of biochemical activity but because of adverse effects and a lack of clinical benefit. Phase II/III BACE1 inhibitors that have been discontinued are selected for repurposing due to their well-established pharmacokinetic characteristics, verified central nervous system penetration, and proven human target engagement. The high potency and structural optimization of these compounds offer a useful starting point for repositioning within a multitarget therapeutic framework, although they were unable to achieve clinical benefit as monotherapies due to side effects or limited cognitive enhancement. Repurposing clinically advanced BACE1 inhibitors presents a strategic opportunity to leverage previously unidentified off-target interactions with key regulatory proteins involved in autophagy, apoptosis, proteostasis, and neuroinflammation, thereby addressing disease complexity beyond amyloid-centric approaches, given the multifaceted nature of Alzheimer’s disease. Alternative exploration of other targets could yield the long-anticipated relative treatment for AD. Therefore, this study aims to examine the molecular mechanisms underlying the therapeutic effects of discontinued BACE1 inhibitors through drug repurposing, utilizing network pharmacology, molecular docking, and protein-drug interaction predictions.

## 2. Results and Discussion

**Figure 1:**
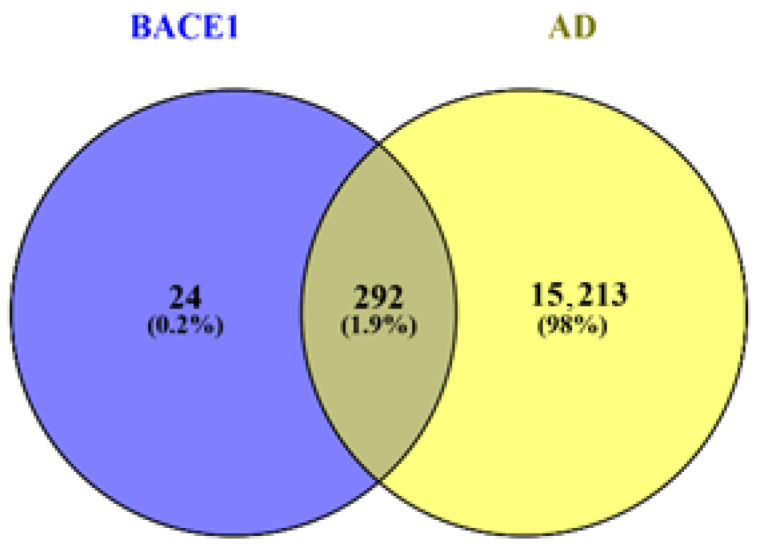
A Venn diagram of BACE1 and AD common proteins.

A Venn diagram of BACE1 inhibitors and AD identified 292 common proteins, as shown in Figure 1. As depicted in Figure 1, 24 (0.2%) unique protein targets for BACE1 that are not associated with AD, marked in the blue region. There are 15,214 (98%) specific AD proteins not linked to BACE1, shown in the yellow region. The 1.9% (292) common proteins linked to BACE1 and AD indicate shared treatment targets, therefore constitute the primary focus for further investigation, including PPI, KEGG, GO, molecular docking, and protein–ligand interaction analysis.

**Figure 2:**
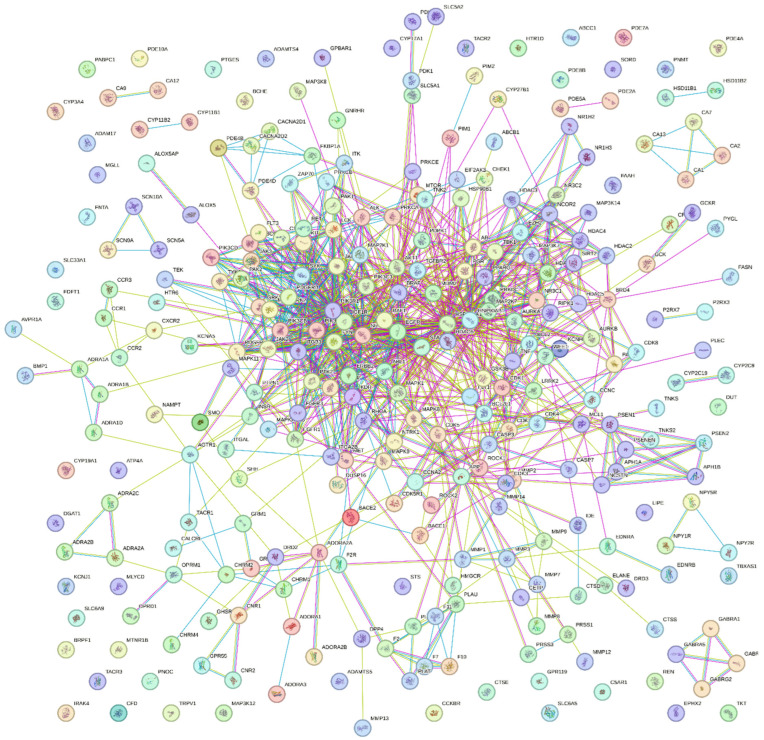
Protein–Protein Interaction Network for BACE1-AD.

Further investigation into BACE1-AD-associated biological processes is made easier by the PPI network, as depicted in Figure 2 below, which illustrates BACE1-AD interactions with a high degree of certainty. A highly dense, critical protein at the centre, with widely dispersed nodes, is revealed by the significant connectivity of the many networks. The PPI reveals that BACE1 and BACE2 are among the common protein targets predicted to be involved in AD pathogenesis, as previously reported in the literature [8]. Hub gene identification may disclose the enrichment analysis of GO and KEGG pathways, necessitating more research.

**Figure 3:**
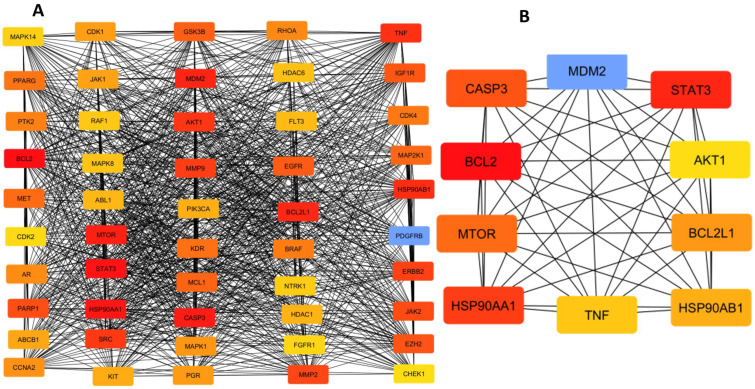
Representation of AD 50 hub proteins (**A**), and 10 hub proteins (**B**).

For the identification of hub proteins from the 292 BACE1-AD common proteins, an initial prediction of fifty (50) was computed using the CytoHubba app in Cytoscape 3.10.3, employing the Maximal Clique Centrality (MCC) and Degree topological analytic methods. The MCC is used to find the largest cliques. A gene or protein node is crucial for network stability and function, indicating that it is a vital biological process. It is quite sensitive and precise when it comes to identifying hub genes in highly dense and coupled networks. Degree centrality, then, is the direct number of connections between various nodes. The direct connections improve with the number of nodes. Degrees are employed in preliminary selection, but because they occasionally leave out crucial context specificity, they are less accurate and sensitive than MCC [9,10,11]. Surprisingly, BACE1 and BACE2 were not among the 50 and 10 hub proteins predicted in the Cytoscape, as illustrated in Figure 3A,B. This suggests that BACE1 and BACE2 are not the primary protein targets in the hallmark of AD and could provide a reason why there are no FDA-approved BACE1 drugs.

The identified BACE1-AD 10 hub proteins include Caspase-3 (CASP3), Signal Transducer and Activator of Transcription 3 (STAT3), B-cell Lymphoma 2 (BCL2), AKT Serine/Threonine Kinase 1 (AKT1), Mechanistic Target of Rapamycin (MTOR), BCL2-Like 1 (BCL2L1), Heat Shock Protein 90 Alpha Family Class A Member 1 (HSP90AA1), Heat Shock Protein 90 Beta Family Member 1 (HSP90AB1), Tumour Necrosis Factor (TNF), and Mouse Double Minute 2 Homolog (MDM2) depicted in Figure 3B. These hub proteins, their functions, and implications in AD pathogenesis are depicted in Table 1 below.

Using the FDR cut-off (0.05), the minimum pathway size (2), and the maximum pathway size (2000), the 10 most critical AD pathways were computed. In this study, under the Pathway database menu, all available gene sets, including GO biological process, GO cellular component, GO molecular function, and KEGG, are chosen and analysed separately. The findings are discussed and presented in Table 2 below and further illustrated in the Supplementary Figures S1–S5.

The KEGG enrichment analysis of the proteins linked to AD (CASP3, STAT3, BCL2, AKT1, MTOR, BCL2L1, HSP90AA1, HSP90AB1, TNF, and MDM2) shows a notable over-representation of pathways associated with apoptosis, neuroinflammation, proteostasis, vascular dysfunction, and infection processes that are increasingly acknowledged as being crucial to the pathogenesis of AD. The very low FDR values (10^−9^–10^−13^) and high fold-enrichment scores (45–155×) indicate that the enriched pathways are statistically significant and not due to random chance. This shows that the AD-associated proteins cluster strongly within a small set of biologically meaningful processes. These data indicate that these pathways, especially PI3K–AKT–MTOR signalling, apoptosis, neuroinflammation, lipid dysregulation, and viral-response pathways, are not minor but rather major molecular processes that contribute to the development of AD. The dysregulation of AKT1 and MTOR in AD has been previously reported in the literature [41,42]. The significance of mitochondrial apoptosis in AD, where elevated CASP3 activation and BCL2/BCL2L1 imbalance contribute to progressive neuronal loss, as previously reported in the literature, is reflected in the enrichment of apoptosis-associated pathways, such as platinum treatment resistance and cancer-related signalling [43,44]. Consequently, HSP90 proteins stabilize tau kinases and promote the build-up of hyperphosphorylated tau, a characteristic feature of AD. Therefore, the presence of HSP90AA1 and HSP90AB1 within these pathways further emphasizes proteostatic failure [45].

Table 2 further illustrates the significant enrichment of neuroinflammatory and cytokine-driven pathways, including TNF signalling, STAT3 activation, and infectious disease pathways such as Epstein–Barr virus (EBV) infection and herpes simplex virus 1 (HSV-1) infection. Highly relevant and fold-enriched viral pathways were identified, which is consistent with previous findings indicating that persistent neuroinflammation, Aβ overproduction, and neuronal death are caused by chronic viral reactivation, especially HSV-1 [46]. Enriched in several pathways, TNF-α and STAT3 are known mediators of astrocyte reactivity, microglial activation, and synaptic dysfunction, all of which contribute to the acceleration of cognitive loss in AD [15,36,47]. Thus, the identification of infectious illness pathways supports the neuroinflammation-infection theory of AD by indicating that these host-response genes are involved in both chronic neurodegenerative cascades and antimicrobial signalling [48].

AKT1, MTOR, TNF, and STAT3 are important regulators of lipid metabolism, inflammatory vascular remodelling, and endothelial homeostasis. Therefore, their enrichment is in line with clinical findings showing cerebrovascular dysfunction, dyslipidaemia, and atherosclerosis greatly raise the risk of AD and worsen Aβ accumulation [49]. Proteoglycans in cancer is another considerably enriched pathway. When combined, these KEGG findings provide a comprehensive systems-level understanding of how AD pathogenesis is driven by apoptosis, impaired autophagy, neuroinflammation, vascular dysfunction, and viral triggers. The involvement of these pathways emphasizes that AD is a multifaceted network disorder that integrates metabolic stress, proteostatic failure, immune system dysfunction, and other factors, rather than being caused by a single molecular malfunction.

**Figure 4:**
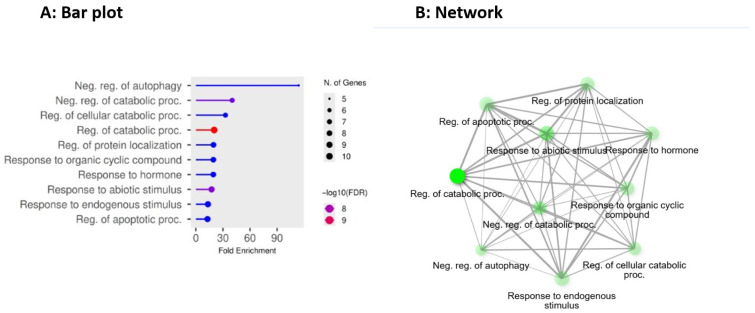
(**A**,**B**) depict Gene Ontology (GO) biological process enrichment analysis of the ten BACE1-AD hub proteins. The bar plots highlight significantly enriched processes related to negative regulation of autophagy, regulation of catabolic processes, and regulation of apoptotic pathways, indicating impaired protein clearance, defective cellular recycling, and apoptosis as central mechanisms in Alzheimer’s disease. These results emphasize autophagy-apoptosis imbalance as a major functional consequence of the identified multitarget network.

Analysis of the GO biological process includes the negative regulation of autophagy, negative regulation of catabolic process, regulation of cellular catabolic process, regulation of catabolic process, regulation of protein localization, response to organic cyclic compound, response to hormone, response to abiotic stimulus, response to endogenous stimulus, and regulation of apoptotic process, as illustrated in Figure 4A,B. The GO biological process reveals some pivotal pathways in sustaining neural proteostasis and organelle quality control, encompassing the regulation and inhibition of autophagy, as well as extensive catabolic pathways and cellular catabolic processes [50]. Basal autophagy in healthy neurons eliminates misfolded proteins, damaged organelles, and other cellular waste, preventing the accumulation of aggregates and maintaining cellular balance. In AD, there is a disruption of these catabolic processes, including reduced expression of critical autophagy-lysosomal pathway components such as Beclin-1, crucial ATG proteins, and improper autophagosome fusion or formation, as reported in the literature [51]. This leads to the accumulation of toxic molecules that cause neuronal stress and degeneration, such as misfolded or aggregated Aβ, hyperphosphorylated tau, and damaged mitochondria [52]. Additionally, autophagy dysregulation and apoptosis regulation are linked. For example, anti-apoptotic Bcl-2 family members bind to Beclin-1, suppressing autophagy initiation and tilting the balance toward cell death rather than survival or cleaning [53]. The GO biological process further underlines how deficiencies in the negative control of autophagy, the regulation of catabolic processes and protein localization, and the regulation of apoptotic processes intersect to promote AD pathogenesis.

Moreover, neuronal responses to endogenous and external stimuli, including oxidative stress, metabolic imbalance, or inflammatory signals, often trigger adaptive alterations in autophagy, protein trafficking, and subcellular protein localization. Consequently, GO biological process pathways, such as response to organic cyclic compounds, hormones, abiotic stimuli, and endogenous stimuli, reflect this larger regulatory potential. Reactive oxygen species, neuroinflammation, metabolic dysfunction, and other stresses exacerbate autophagy impairment, lysosomal dysfunction, and protein localization disruption in AD, thereby increasing aggregate formation and organelle damage [54,55]. A recent study by Zhou et al. also shows that restoring appropriate autophagy and catabolic flux can alleviate Aβ and tau pathology, reduce neuroinflammation, and sometimes improve cognitive outcomes in animal models [56]. This can be achieved through the selective degradation of damaged mitochondria (mitophagy), the enhancement of autophagosome-lysosome fusion, or the modulation of upstream signalling, including MTOR [56]. Collectively, these results support the biological relevance of GO processes in controlling autophagy, catabolism, protein localization, and cellular stress responses as essential mechanisms in AD, and support their application as functional annotation for evaluating AD-related gene sets.

**Figure 5:**
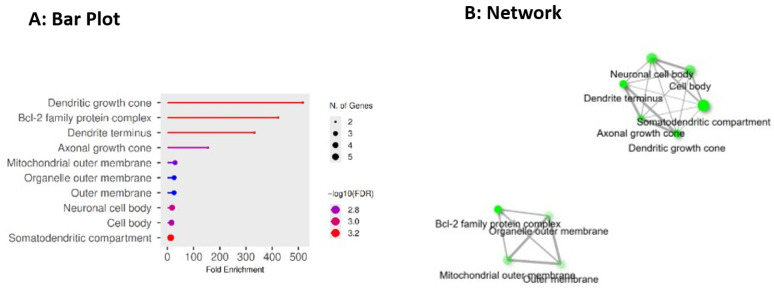
(**A**,**B**) depict GO cellular component enrichment analysis showing the subcellular localization of BACE1-AD hub proteins. Enrichment in mitochondrial outer membrane, Bcl-2 family protein complexes, somatodendritic compartments, and axonal/dendritic growth cones underscores the involvement of mitochondrial dysfunction, synaptic integrity loss, and compartment-specific neuronal degeneration in Alzheimer’s disease pathology. Bottom of Form.

GO Cellular Components include dendritic growth cone, Bcl-2 family protein complex, dendrite terminus, axonal growth cone, mitochondrial outer membrane, organelle outer membrane, outer membrane, neuronal cell body, cell body, and somatodendritic compartment as illustrated in Figure 5A,B below.

Cellular compartment impairment that modulates neuronal connectivity, such as dendritic and axonal growth cones, dendrite termini, somatodendritic compartments, and neuronal cell bodies, plays a key role in AD. Early synaptic failure is triggered by the disruption of the growth cone and dendritic compartment. Tau-mediated cytoskeletal instability and abnormal actin/microtubule remodelling hinder spine formation and maintenance, collapsing the structural underpinning for plasticity and memory. These compartmental deficits are associated with quantifiable markers of axonal and synaptic injury and are linked to increasing cognitive loss, demonstrating that disease at growth cones and dendritic termini represents an upstream, mechanistic step in AD neurodegeneration [57]. A pathological link exists between compartmental structural collapse and neuron degeneration, which is mediated by the outer mitochondrial membrane’s molecular machinery, including the Bcl-2 family protein complex, that regulates mitophagy and apoptotic processes [58]. In susceptible neurons, disrupted Bcl-2/BCL2L1 equilibrium drives mitochondrial outer-membrane permeabilization, impaired proteostasis, and bioenergetic collapse. Collectively, these processes worsen somatodendritic and axonal pathology in AD. Furthermore, it regulates caspase-driven apoptosis and non-canonical functions in mitophagy/autophagy [59]. Chernyuk et al. reported that Bcl-2-regulated outer-membrane processes are intriguing targets for retaining neuronal soma and processes in AD, because compartmentalized mitophagy and cardiolipin-mediated outer-membrane signalling are crucial factors in determining whether damaged mitochondria are cleared or cause cell death [60]. Therefore, targeting these hub proteins including CASP3, STAT3, BCL2, AKT1, MTOR, BCL2L1, HSP90AA1, HSP90AB1, TNF, and MDM2 suggests promising drug discovering route for AD.

**Figure 6:**
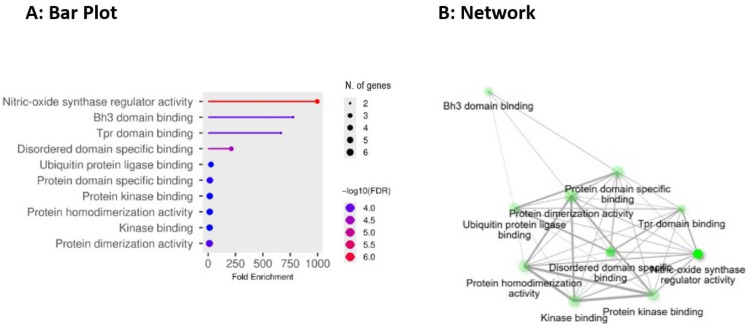
(**A**,**B**) depict GO molecular function enrichment analysis of BACE1-AD hub proteins. Prominent functions include BH3-domain binding, kinase binding and dimerization, ubiquitin ligase binding, and interactions with chaperone-associated domains, reflecting the coordinated regulation of apoptosis, autophagy, protein quality control, and inflammatory signalling. These functions support a multitarget, network-driven mechanism underlying Alzheimer’s disease progression.

Lastly, GO Molecular Function includes nitric-oxide synthase regulator activity, BH3 domain binding, tetratricopeptide repeat domain binding (TPR) domain binding, disordered domain-specific binding, ubiquitin protein ligase binding, protein domain-specific binding, protein kinase binding, protein homodimerization activity, kinase binding, and protein dimerization activity as illustrated in Figure 6A,B below.

The GO molecular function results overlap significantly with established mechanistic pathways in Alzheimer’s disease (AD). The apoptosis/mitophagy switch and mitochondrial outer-membrane permeabilization are directly controlled by BH3-domain binding, such as BCL2/BCL2L1. An imbalance of connections between anti- and pro-apoptotic BCL-2 family members at the mitochondrial outer membrane enhances cytochrome c release, caspase activation, and neuronal death in AD models [61]. TPR-domain and disordered-domain binding indicate an interaction between HSP90/HSP70 chaperones (HSP90AA1/HSP90AB1) and co-chaperones, as well as pathogenic client proteins such as tau and kinases. Moreover, TPR-mediated interactions stabilize tau-kinase complexes and proteotoxic species, thereby hindering their removal and promoting the accumulation of these species, which in turn leads to synaptic damage [62]. Ubiquitin-protein ligase binding, such as MDM2, and stronger ubiquitin-proteasome interactions, attract an additional vital axis. Reduced E3 ligase activity and proteasomal dysfunction are commonly found in AD and can modify p53 signalling, tau ubiquitination, and downstream neuronal survival choices, thereby linking protein quality control failure to neurodegeneration [63].

Consequently, nitric oxide (NO) synthase regulator activity, believed to be regulated by cytokines and kinases such as TNF and AKT, reflects how inflammatory and metabolic signalling adjusts NO synthesis. Dysregulated NO signalling leads to oxidative damage and synapse loss in AD, indicating NO’s dual function in both synaptic physiology and neurotoxicity [64]. In addition to these interactions, the GO functions for protein/kinase binding and dimerization (AKT1, MTOR, STAT3, CASP3) highlight signalling pathways that regulate survival, phosphorylation cascades, and proteolysis. AKT/MTOR kinase interactions regulate autophagy and protein trafficking, while MTOR hyperactivity inhibits autophagic clearance of Aβ/tau. Moreover, STAT3 dimerization promotes transcriptional programs of neuroinflammation; both processes are sustained by HSP90 chaperone complexes that bind kinases and alter their activity in AD models [42]. Caspase-3’s protease activity and its regulation via dimerization/activation offer a molecular pathway for tau breakage and synapse degeneration, enabling a proteolytic path from signalling failure to the conventional symptoms of AD [65]. Collectively, these molecular-function annotations, including BH3 binding, TPR-mediated chaperone interactions, E3 ligase engagement, kinase binding/dimerization, and NO regulation, provide an integrated functional framework in which impaired proteostasis, maladaptive signalling, inflammation, and regulated cell death combine to drive AD. They also offer feasible molecular pathways for treatment modification.

Repurposing the failed BACE1 inhibitors, including Verubecestat, Lanabecestat, Elenbecestat, Umibecestat, and AM-6494 focusing on non-BACE1 targets such as CASP3, STAT3, BCL2, AKT1, MTOR, BCL2L1, HSP90AA1, HSP90AB1, TNF, and MDM2, offers a logical and promising approach considering the key involvement of these hub proteins in neurodegeneration, inflammatory responses, and proteostasis. Considering that these BACE1 inhibitors lacked clinical breakthroughs due to limited cognitive improvements and adverse reactions, their characterized pharmacokinetics and safety profiles provide a solid basis for repurposing in a multitarget AD approach. Concurrently, several of these identified hub proteins are critical controllers of neuron viability and apoptosis. CASP3 induces apoptosis, while BCL2/BCL2L1 regulates mitochondrial membrane integrity. AKT1 and MTOR regulate autophagy and protein synthesis [13,19,22,25,27]. Moreover, STAT3 and TNF are crucial in chronic brain inflammation, a known and acknowledged contributor to AD progression. Furthermore, MDM2 regulates p53-mediated neuronal stress responses, while HSP90AA1/AB1 contributes to tau stability and protein folding. Monofunctional amyloid-focused medications could become multifunctional neuroprotective agents by structurally altering and optimizing these BACE1 scaffolds in silico to increase affinity. These repurposed drugs are promising prospects for next-generation therapy development, as this polypharmacological approach aligns with current data indicating that AD is a complex illness that requires extensive pathway regulation, rather than single-target therapies [15,30,33,36,39].

Molecular docking results (kcal/mol) of the discontinued BACE1 inhibitors with BACE1 protein and non-BACE1 targets such as CASP3, STAT3, BCL2, AKT1, MTOR, BCL2L1, HSP90AA1, HSP90AB1, TNF, and MDM2 are illustrated in Table 3 below.

A distinct change from a single-target to a multi-target therapeutic approach is revealed by molecular docking analysis (Table 3) of the five unsuccessful BACE1 inhibitors (Verubecestat, Lanabecestat, Elenbecestat, Umibecestat, and AM-6494) against the network pharmacology-identified targets CASP3, STAT3, BCL2, AKT1, MTOR, BCL2L1, HSP90AA1, HSP90AB1, TNF, and MDM2. Among all the BACE1 inhibitors, Umibecestat showed the greatest binding to AKT1 (−11.4 kcal/mol), followed by Lanabecestat (−10.6 kcal/mol) and Verubecestat (−10.3 kcal/mol), suggesting an exceptionally high binding affinity for the PI3K/AKT survival pathway. Verubecestat demonstrated one of the most significant bindings to HSP90AB1 (−10.3 kcal/mol), indicating that it could be useful in regulating proteostasis and clearing misfolded proteins, a key pathological feature of AD. All three compounds also demonstrated highly favourable binding to the molecular chaperones HSP90AA1 and HSP90AB1.

On the other hand, whereas binding to anti-apoptotic and inflammatory targets, such as BCL2, BCL2L1, STAT3, and TNF, was fairly high, it was consistent among compounds, indicating their ability to collectively control neuroinflammation and apoptosis. Essentially, despite the fact that the medications were initially designed to target BACE1, the docking results reveal that several inhibitors now exhibit binding affinities to non-BACE targets that are equal to or higher than those to BACE1 itself. For example, Lanabecestat (−7.6 kcal/mol to BACE1) and Verubecestat (−8.9 kcal/mol to BACE1) showed stronger predicted interactions with AKT1 and HSP90 isoforms than with the initial target.

The justification for the repurposing of unsuccessful BACE1 inhibitors as multi-target disorder-modifying drugs for AD, as opposed to their abandonment due to single-target clinical failure, is well-supported by these data. Since it is now commonly known that protein misfolding, defective autophagy, oxidative stress, apoptotic dysregulation, and chronic neuroinflammation all contribute to Alzheimer’s pathology, network-centered therapy methods are preferable over conventional “one drug–one target” approaches. The potential of these drugs to affect several disease-associated mechanisms, such as tau clearance, neuronal survival, and synaptic resilience, is suggested by the high-affinity interactions seen with HSP90, AKT1, and MTOR.

Moreover, interactions with STAT3 and TNF suggest further anti-inflammatory potential, while regulation of BCL2/BCL2L1 and CASP3 suggests possible neuroprotective effects through decreased apoptosis. In complicated neurodegenerative disorders like AD, multitarget medications that may concurrently fix disrupted signalling networks are more likely to be effective, according to recent studies [66]. The molecular docking results thus provide a compelling, biologically plausible, and translationally appealing argument for repurposing BACE1 inhibitors as novel lead candidates in AD drug discovery, when paired with the pharmacokinetic and blood–brain barrier data currently available for these compounds from prior clinical trials. The protein–ligand interaction (Supplementary Figures S6–S11 and Table 4) further reveals the protein active site residues involved in binding, the hydrogen bond network, and the ionic and hydrophobic interactions that contribute to a stable complex.

## 3. Materials and Methods

### 3.1. Compound Screening and Preparation

SMILES of Verubecestat, lanabecestat, elenbecestat, and umibecestat (CNP-520) discontinued at Phase II/III clinical trials, and AM-6494, at the preclinical trial, were obtained from PubChem. The prediction of these inhibitors targeted proteins was computationally computed using the Swiss Target Prediction database [67,68,69]. A total of 5000 protein targets were obtained for the 5 BACE 1 inhibitors. After the data cleaning, 316 proteins were subsequently saved in an Excel spreadsheet. Human proteins associated with the AD were predicted and obtained from GeneCards, a database that has all the information on all the gene sets that are linked to diseases [70]. A total of 15,506 proteins (genes) were obtained, cleaned, and subsequently saved in an Excel spreadsheet for further screening on the VENNY webpage. The computation of common proteins in VENNY 2.1.0 generated a Venn diagram showing the intersecting proteins for BACE 1 inhibitors and AD [71].

### 3.2. Protein–Protein Interaction (PPI) Network and Hub Genes Analysis

Following the analysis of common proteins, the Protein–Protein Interaction (PPI) network was computed using the Search Tool for the Retrieval of Interacting Genes (STRING) database. The PPI displays the interactions with a high degree of confidence for further analysis [72]. After selecting Homo sapiens from the drop-down menu and choosing the multiple proteins icon in the STRING database, the common genes were uploaded, and the PPI network was calculated. High-resolution Portable Network Graphics (PNG) were used to export and store the analyzed PPI network. The PPI network was also sent to Cytoscape to find hub genes [73]. The hub genes are sometimes referred to as key genes that are involved in relevant biological processes, ensuring the stability of the PPI network. They reveal the mechanistic dynamics of diseases by controlling cell functioning. Cytoscape 3.10.3, the most recent version, was downloaded. After that, yfile and Cytohubba were included in the application. To identify hub genes, the PPI network was exported from STRING and opened in Cytohubba in Cytoscape. To rank the hub genes according to centrality and relevance in the PPI network, a selection of hub genes was made using the MCC and degree topological analysis methodologies.

### 3.3. Functional Enrichment Analysis

The ShinyGO 0.85 database was utilized for Kyoto Encyclopaedia of Genes and Genomes (KEGG) and the Gene Ontology (GO) pathway analysis [74]. The hub genes were added to the database and examined to determine their functional enrichment, which enabled the understanding of their biological roles, identification of potential therapeutic targets, and elucidation of the mechanisms underlying AD [75,76]. The computation of *p*-values and False Discovery Rates (FDR) is performed using the hypergeometric test and the Benjamini–Hochberg technique [77]. The fold enrichment is computed as the percentage of genes in the 10 hub genes that are in a pathway divided by the corresponding percentage in the background genes. The fold enrichment is a direct measurement of the effect size, while FDR indicates the statistical significance [78]. In this study, an FDR cut-off of 0.05 was used to show the 10 most useful pathways. The average sorting of the pathways is done using FDR and fold enrichment when ‘Select by FDR and Sort by Enrichment’ was selected.

### 3.4. Protein and Ligand Preparations

The X-ray crystalized structures of the 10 hub genes/proteins were retrieved from the Research Collaboratory for Structural Bioinformatics Protein Data Bank (RCSB PDB) with PDB IDs 3DEI (CASP3), 6NUQ (STAT3), 1PBK (MTOR), 4R3M (HSP90AA1), 3LBK (MDM2), 7JRA (TNF), 4QVX (BCL2L1), 1UYM (HSP90AB1), 3O96 (AKT1), and 4AQ3 (BCL2) [79]. Furthermore, Protein preparation in PyRx 0.8 was carried out, which involves importing the target protein structure, eliminating any bound ligands and water molecules, adding polar hydrogens, allocating Gasteiger charges, and storing the structure in the AutoDock Vina 1.1.2-compatible PDBQT format. To prepare the 5 BACE1 ligands, each was optimized in its geometry through energy minimization, all hydrogens were added, charges were assigned, rotatable bonds were defined for flexibility, and the ligand was exported as a PDBQT file [80].

### 3.5. Molecular Docking and Protein–Ligand Interaction Analysis

Molecular docking was computed using PyRx to predict and evaluate binding energies for the predicted BACE1 inhibitors and non-BACE1 hub proteins. Molecular docking reveals the likely multi-targeted molecular processes of the complexes that consistently align with the network pharmacology structure [80]. Molecular docking further reveals multiple therapeutic targeted networks rather than single-targeted ones. Molecular docking was performed using AutoDock Vina 1.1.2 implemented within the PyRx virtual screening platform. The PubChem database was used to obtain the three-dimensional structures of the chosen ligands (Verubecestat, Lanabecestat, Elenbecestat, Umibecestat, and AM-6494). These structures were then loaded into PyRx, where ligand geometry optimization and energy minimization were carried out using Open Babel 2.3.2 with the universal force field (UFF). prior to docking and optimization, water molecules and co-crystallized ligands were eliminated from the target protein structures that were acquired from the Protein Data Bank. The Vina Wizard was used to construct docking grids that included the active or regulatory binding domains of each target protein. The binding affinities were reported as binding free energy values (kcal/mol) after molecular docking was performed using AutoDock Vina with default exhaustiveness parameters. To examine hydrogen bonding, hydrophobic, and π-alkyl interactions, docking postures were selected based on the lowest binding energy [80,81,82]. Protein–ligand interactions computed with BIOVIA Discovery Studio software v21.1.0.20298 reveal the protein active site residues involved in binding, the hydrogen bond network, and the ionic and hydrophobic interactions responsible for a stable and specific protein–ligand complex, highlighting the synergistic roles in inhibiting AD progression [83,84,85].

## 4. Conclusions

AD is neuropathologically defined by the formation of neurofibrillary tangles and β-amyloid plaques, and for nearly two decades, BACE1 has remained a dominating treatment target. Although several small-molecule BACE1 inhibitors, including verubecestat, lanabecestat, elenbecestat, umibecestat (CNP-520), and AM-6494, entered clinical development, their eventual discontinuation highlighted the drawbacks of a single-target approach in multifactorial disorders like AD. By methodically examining the repurposing potential of these unsuccessful BACE1 inhibitors using an integrative computational framework that incorporates network pharmacology, molecular docking, and protein–ligand interaction research, the current study offers a compelling alternative perspective. Key AD-related hub proteins, including CASP3, STAT3, BCL2, AKT1, MTOR, BCL2L1, HSP90AA1, HSP90AB1, TNF, and MDM2, showed a marked over-representation of pathways associated with apoptosis, neuroinflammation, poor proteostasis, vascular dysfunction, and infection, according to KEGG enrichment analysis. These molecular mechanisms serve as fundamental causes of neurodegeneration and are consistent with new theories that view AD as a complex, network-based illness rather than a pathology that is solely focused on amyloid. The significance of these targets was further supported by Gene Ontology enrichment, which showed high correlations with dysregulated autophagy, modified catabolic processes, apoptotic imbalance, and abnormal signalling responses to endogenous, hormonal, and abiotic stimuli. At the cellular level, these proteins are primarily found in structures closely related to the survival and integrity of neurons, including Bcl-2 family protein complexes, somatodendritic compartments, the mitochondrial outer membrane, and growth cones of dendrites and axons. Therefore, more in silico, in vitro, and in vivo validations are needed to validate these results. Future studies should prioritize mechanistic validation using neuronal and glial cell-based assays to assess autophagic flux (LC3-II, p62, Beclin-1) and AKT–MTOR signalling following treatment with repurposed BACE1 inhibitors. Additional in vitro assays examining HSP90-mediated proteostasis, caspase-3 activation, and BCL2-family-regulated apoptosis will further substantiate the predicted multi-target mechanisms. Neuroinflammatory modulation should be evaluated in microglial and astrocytic models via STAT3 activation and TNF-α release. Finally, in vivo validation using transgenic Alzheimer’s disease mouse models, combined with behavioural cognition assays and molecular neuropathology endpoints, will be essential to establish translational relevance and therapeutic potential.

## Figures and Tables

**Table 1:** BACE1-AD Hub Proteins, their functions, and implications.

Common Name	Full Name	Function	Association	Clinical Relevance	References
CASP3	Caspase-3	A protein that regulates apoptosis, when important proteins in the cell are cleaved.	Very active in AD brains; causes cell death, Aβ-induced toxicity, tau cleavage, and the creation of neurofibrillary tangles. Active caspase-3 is significantly elevated in post-mortem AD brains and colocalizes with neurofibrillary tangles and dystrophic neurites. Caspase-3 promotes Aβ-induced synaptic loss and tau cleavage in human neurons.	Caspase-3 activation correlates with synapse degradation rather than late-stage neuronal death, linking it directly to cognitive decline.	[12,13,14]
STAT3	Signal Transducer and Activator of Transcription 3	A transcription factor that controls how cells survive, respond to inflammation, and fight infections	Aberrant STAT3 activation exacerbates neuronal inflammation and the astrocyte response. In AD models, STAT3 in glia lowers Aβ accumulation and improves cognition.	In AD, STAT3 signaling has a role in both cytokine amplification and reactive astrogliosis.	[15,16,17]
BCL2	B-cell Lymphoma 2	Anti-apoptotic protein that stops the release of cytochrome c from mitochondria and cell death	Lowering BCL2 levels makes neurons more sensitive to Aβ and oxidative stress. Higher BCL2 is protective and may slow down the death of neurons.	The hippocampi of AD patients exhibit altered BCL2 balance, which is linked to increasing neuronal death.	[17,18,19,20]
AKT1	AKT Serine/Threonine Kinase 1	Central kinase in PI3K/AKT signalling that helps cells stay alive, grow, and use energy	Impaired AKT signalling leads to neuronal disorder, tau hyperphosphorylation, and Aβ toxicity.	AKT signaling is impaired in AD patients, according to CSF investigations and post-mortem hippocampus analysis.	[21,22,23]
MTOR	Mechanistic Target of Rapamycin	The main controller of cell growth, metabolism, and autophagy.	When MTOR is overly active, it inhibits autophagy, making it more difficult to eliminate Aβ and phosphorylated tau.	Elevated MTOR signaling correlates with amyloid burden and cognitive decline in AD patients.	[24,25,26]
BCL2L1	BCL2-Like 1	Mitochondrial protein that stops cytochrome-c from being released.	BC2L1 safeguards neurons from Aβ-induced apoptosis. Dysregulation leads to mitochondrial impairment and neuronal degeneration in Alzheimer’s disease.	The mitochondrial dysfunction seen in AD patient neurons is partly caused by BCL2L1 dysregulation.	[18,27,28]
HSP90AA1	Heat Shock Protein 90 Alpha Family Class A Member 1	A molecular chaperone that helps proteins fold, stay stable, and respond to stress.	HSP90 increases tau hyperphosphorylation by stabilizing tau kinases such as CDK5 and GSK-3β. Tau aggregates are decreased when HSP90 is inhibited.	In human-derived neuronal models, tau pathology is decreased by pharmacological suppression of HSP90AA1.	[29,30,31]
HSP90AB1	Heat Shock Protein 90 Beta Family Member 1	Protein folding and breakdown are aided by the constitutive cytosolic HSP90 isoform.	Proteotoxic stress causes it to be upregulated in AD; it interacts with tau and helps keep it stable. Tau pathology is decreased by targeting HSP90. The constitutive cytosolic HSP90 isoform, HSP90AB1, interacts directly with pathogenic tau species and exhibits region-specific increase in the hippocampus of AD patients.	Human post-mortem investigations show that HSP90AB1 is distributed differently across the phases of AD, suggesting that it is related to the advancement of the disease rather than overall proteostasis.	[31,32,33,34]
TNF	Tumour Necrosis Factor	Pro-inflammatory cytokine, essential for cell death and immune activation processes	Synaptic dysfunction, Aβ generation, and chronic neuroinflammation are all driven by elevated TNF-α in AD. In several models, anti-TNF therapies have been shown to improve cognitive function.	Anti-TNF treatments have demonstrated cognitive benefits in observational studies, and elevated TNF-α levels are correlated with cognitive impairment.	[35,36,37]
MDM2	Mouse Double Minute 2 Homolog	E3 ubiquitin ligase, which controls cell development/apoptosis and p53 stability.	Neuronal death as a result of altered MDM2-p53 signalling. AD is associated with p53 accumulation (caused by decreased MDM2 activity), which stimulates apoptosis in response to Aβ.	Human neurodegenerative diseases, such as AD, have been shown to exhibit dysregulation of the MDM2–p53 axis, which links compromised proteostasis to neuronal death.	[38,39,40]

**Table 2:** Ten (10) top AD pathways selected and sorted by FDR and Fold Enrichment.

Enrichment FDR	nGenes	Pathway Genes	Fold Enrichment	Pathways (Click for Details)
1.5 × 10^−9^	5	75	155.1	Platinum drug resistance
1.5 × 10^−9^	5	79	147.2	EGFR tyrosine kinase inhibitor resistance
4.9 × 10^−11^	6	97	143.9	Prostate cancer
8.4 × 10^−11^	6	111	125.7	Toxoplasmosis
3.3 × 10^−13^	8	215	86.5	Lipid and atherosclerosis
1.1 × 10^−9^	6	181	77.1	Herpes simplex virus 1 infection
1.5 × 10^−9^	6	202	69.1	Epstein-Barr virus infection
1.5 × 10^−9^	6	203	68.7	Proteoglycans in cancer
7.6 × 10^−10^	7	362	45	PI3K-Akt signalling pathway
1.2 × 10^−12^	9	529	39.6	Pathways in cancer

**Table 3:** Molecular docking results of BACE1 inhibitors and the hub proteins showing the binding energy in kcal/mol.

BACE1 Inhibitors	BACE1	CASP3	STAT3	BCL2	AKT1	MTOR	BCL2L1	BCL2L1	HSP90AA1	HSP90AB1	TNF	MDM2
Verubecestat	−8.9	−7.5	−8.6	−7.5	−10.3	−7.7	−7.5	−8.4	−8.3	−10.3	−6.9	−7.7
Lanabecestat	−7.6	−8.5	−7.5	−7.4	−10.6	−7.3	−7.4	−9.2	−7.5	−9.9	−7.5	−8.2
Elenbecestat,	−10.1	−7.4	−8.2	−7.5	−9.5	−7.6	−7.5	−10.6	−8.5	−9.3	−7.5	−7.5
Umibecestat	−10.4	−7.5	−8.9	−8.5	−11.4	−8.3	−8.5	−8.8	−8.5	−9.5	−7.3	−7.3
AM-6494	−8.5	−6.6	−6.8	−7.1	−7.4	−6.8	−6.9	−7.5	−7.1	−9.5	−6.8	−6.8
Cocrystallized	10.2	−8.2	−9.0	−11.0	−14.7	−7.6	−10.9	−9.2	−11.0	−7.9	−7.5	−8.6

**Table 4:** Protein–ligand interaction analysis of the BACE1 inhibitor complexes with BACE1 and non-BACE1 proteins.

Proteins	Ligands	Interacting Residues & Type of Bonds	Implications in AD
AKT1	Umibecestat	TYR67, ASP70, GLN77, PHE63, PHE71, VAL92, LEU96, LEU78, MET74, ARG88, ALA108, THR91, and GLU95. Van der Waals, Pi-Alkyl, Alkyl, and Halogen (F)	AKT1 signalling may be modulated or inhibited by interactions within kinase activity-related areas. Umibecestat binds with AKT1, supporting a repurposing rationale that involves decreased tau pathology and enhanced neuronal quality control in AD, as AKT1 hyperactivation is associated with tau hyperphosphorylation, impaired autophagy, and poor neuronal survival of damaged cells. Binding at these residues is predicted to modulate AKT1 kinase activity rather than nonspecific surface association. Since AKT1 hyperactivation in Alzheimer’s disease is linked to downstream mTOR overactivation, tau hyperphosphorylation, and impaired autophagy, modulation of this region provides a mechanistic basis for the observed association with reduced tau pathology and improved neuronal quality control, thereby supporting the rationale for repurposing.
BACE1	Umibecestat	TYR71, TYR198, VAL332, ILE226, ILE110, TRP115, GLY11, GLN73, THR72, ASP228, THR231, and THR232. Carbon H-bonds, conventional H-bonds, Alkyl, van der Waals, and Pi-Alkyl	Effective enzyme targeting is confirmed by strong multi-point anchoring within the catalytic region of BACE1. High inhibitory stability is suggested by the existence of several hydrogen bonds with catalytic or adjacent residues, such as ASP228 and GLN73, which support decreased amyloidogenic processing of APP.
BCL2L1	Elenbecestat	SER106, GLU129, ASP133, ALA142, ARG102, ASN106, PHE105, PHE146, LEU108, ARG132, PHE131, LEU130, and ALA149. Van der Waals, π–π/π–alkyl, halogen bonding, and H-bonding	Apoptosis-linked AD processes are influenced by the stable engagement of the BH3 groove.
BCL2	Umibecestat	ARG273, GLN79, ASP292, TYR72, TRP80, ILE84, VAL270, LEU264, GLY294, ASN54, GLU298, VAL271, TYR326, LYS268, SER205. Van der Waals, Pi-Alkyl, Alkyl, Halogen (F), and conventional H-bonds	A crucial functional area of BCL2, the BH3-binding groove, contains residues that the ligand occupies. Possible reduction in BCL2’s anti-apoptotic function is indicated by strong hydrogen bonding and aromatic/hydrophobic interactions (TRP80, TYR72, ARG273). This could encourage the removal of malfunctioning or degenerating neurons in AD, thereby reducing the accumulation of toxic proteins and promoting normal neuronal function and survival.
TNF	Lanabecestat	LEU218, TYR217, ASP216, and ALA98. Van der Waals and H-bonding	Inflammation-control measures in AD are supported by effective engagement of TNF.
TNF	Elenbecestat	GLN143, ASP216, PHE220, TYR217, GLY142, LYS141, LEU218, PRO96, GLU99, GLU101. Van der Waals, π–π stacking, halogen bonding, and H-bonding	Reduces neuroinflammatory signalling in AD via stabilizing TNF interactions.
CASP3	Lanabecestat	ARG207, TYR204, LEU168, TRP206, SER209, ASN208, TRP214, PHE256, THR166. Van der Waals, alkyl/π-alkyl, and H-bonding.	promotes the regulation of caspase-3, which is important for neuronal death in AD.
HSP90AB1	Lanabecestat	LYS58, ALA55, PHE138, MET98, ASP54, LEU107, ILE110, THR184, ASN106, and ILE96. Alkyl, van der Waals, and halogen bonding.	Supports crucial proteostasis pathways in AD by stabilizing HSP90AB1
HSP90AA1	Umibecestat	THR515, ARG335, ASP566, ASN567, GLU616, ILE569, ASP570, PRO471, LYS573, MET470, ILE467, and CYS468. Van der Waals, alkyl, halogen bonding, electrostatic, and H-bonding.	Enhances AD-related Hsp90AA1-mediated proteostasis.
MDM2	Umibecestat	TRP162, PHE170, PHE22, LEU107, PHE138, MET98, ALA55, TYR139, LEU103, VAL150, GLY135, ILE110, and ASN51. π–σ, π-alkyl, van der Waals.	Activates MDM2’s hydrophobic regions, which impact AD-related stress-response pathways.
MTOR	Umibecestat	VAL171, ILE172, TYR198, TRP175, PHE216, LEU162, GLU195, LYS170, GLY169, ALA197, and TRP196. Van der Waals, alkyl/π-alkyl, halogen, and H-bonding.	ATP-site stability is in line with Aβ/tau homeostasis and MTOR-regulated autophagy.
STAT3	Umibecestat	LEU54, ILE61, VAL93, TYR67, GLN72, PHE55, MET62, GLN59, ILE103, LEU57, LEU75, GLY58. Van der Waals, π-alkyl, halogen bonding, and H-bonding.	Promotes STAT3 modulation, which is essential for controlling neuroinflammation in AD.

## Data Availability

The original contributions presented in this study are included in the article/Supplementary Material. Further inquiries can be directed to the corresponding authors.

## Supplementary Materials

The following supporting information can be downloaded at https://www.mdpi.com/article/10.3390/ph19010138/s1: Figure S1–S5: Illustration of KEGG Pathways, Figures S6–S11: 2D Protein—Ligand Interaction Complex.

## References

[1] Ugbaja S.C. (2021). Multidimensional Computational Modeling of Potent BACE1 (β-Secretase) Inhibitors towards Alzheimer’s Disease Treatment. Biophys. Chem..

[2] Ugbaja S.C., Lawal I.A., Kumalo H.M., Lawal M.M. (2021). Alzheimer’s Disease and β-Secretase Inhibition: An Update with a Focus on Computer-Aided Inhibitor Design. Curr. Drug Targets.

[3] FDA Approves Treatment for Adults with Alzheimer’s Disease|FDA. https://www.fda.gov/drugs/news-events-human-drugs/fda-approves-treatment-adults-alzheimers-disease.

[4] Sangeet S., Khan A. (2025). Bacopa Monnieri Phytochemicals as Promising BACE1 Inhibitors for Alzheimer’s Disease Therapy. Sci. Rep..

[5] Thomas J., Wilson S. (2024). Molecular and Therapeutic Targets for Amyloid-Beta Plaques in Alzheimer’s Disease: A Review Study. Basic Clin. Neurosci..

[6] Pettus L.H., Bourbeau M.P., Bradley J., Bartberger M.D., Chen K., Hickman D., Johnson M., Liu Q., Manning J.R., Nanez A. (2020). Discovery of AM-6494: A Potent and Orally Efficacious β-Site Amyloid Precursor Protein Cleaving Enzyme 1 (BACE1) Inhibitor with in Vivo Selectivity over BACE2. J. Med. Chem..

[7] Ugbaja S.C., Appiah-Kubi P., Lawal M.M., Gumede N.S., Kumalo H.M. (2021). Unravelling the Molecular Basis of AM-6494 High Potency at BACE1 in Alzheimer’s Disease: An Integrated Dynamic Interaction Investigation. J. Biomol. Struct. Dyn..

[8] Arya R., Jain S., Paliwal S., Madan K., Sharma S., Mishra A., Tiwari P., Kadiri S.K. (2024). BACE1 Inhibitors: A Promising Therapeutic Approach for the Management of Alzheimer’s Disease. Asian Pac. J. Trop. Biomed..

[9] Fang J., Wang X., Xie J., Zhang X., Xiao Y., Li J.K., Luo G. (2023). LGALS1 Was Related to the Prognosis of Clear Cell Renal Cell Carcinoma Identified by Weighted Correlation Gene Network Analysis Combined with Differential Gene Expression Analysis. Front. Genet..

[10] Li C.Y., Cai J.H., Tsai J.J.P., Wang C.C.N. (2020). Identification of Hub Genes Associated With Development of Head and Neck Squamous Cell Carcinoma by Integrated Bioinformatics Analysis. Front. Oncol..

[11] Kumar N., Mukhtar M.S. (2023). Ranking Plant Network Nodes Based on Their Centrality Measures. Entropy.

[12] Putinski C., Abdul-Ghani M., Stiles R., Brunette S., Dick S.A., Fernando P., Megeney L.A. (2013). Intrinsic-Mediated Caspase Activation Is Essential for Cardiomyocyte Hypertrophy. Proc. Natl. Acad. Sci. USA.

[13] Park G., Nhan H.S., Tyan S.H., Kawakatsu Y., Zhang C., Navarro M., Koo E.H. (2020). Caspase Activation and Caspase-Mediated Cleavage of APP Is Associated with Amyloid β-Protein-Induced Synapse Loss in Alzheimer’s Disease. Cell Rep..

[14] Wójcik P., Jastrzębski M.K., Zięba A., Matosiuk D., Kaczor A.A. (2023). Caspases in Alzheimer’s Disease: Mechanism of Activation, Role, and Potential Treatment. Mol. Neurobiol..

[15] Samad M.A., Ahmad I., Hasan A., Alhashmi M.H., Ayub A., Al-Abbasi F.A., Kumer A., Tabrez S. (2025). STAT3 Signaling Pathway in Health and Disease. MedComm.

[16] Wen X., Hu J. (2024). Targeting STAT3 Signaling Pathway in the Treatment of Alzheimer’s Disease with Compounds from Natural Products. Int. Immunopharmacol..

[17] Wen T., Liu T., Chen H., Liu Q., Shen X., Hu Q. (2024). Demethylzeylasteral Alleviates Inflammation and Colitis via Dual Suppression of NF-ΚB and STAT3/5 by Targeting IKKα/β and JAK2. Int. Immunopharmacol..

[18] Vogler M., Braun Y., Smith V.M., Westhoff M.A., Pereira R.S., Pieper N.M., Anders M., Callens M., Vervliet T., Abbas M. (2025). The BCL2 Family: From Apoptosis Mechanisms to New Advances in Targeted Therapy. Signal Transduct. Target. Ther..

[19] Sadoul R. (1998). BCL-2 Family Members in the Development and Degenerative Pathologies of the Nervous System. Cell Death Differ..

[20] Callens M., Kraskovskaya N., Derevtsova K., Annaert W., Bultynck G., Bezprozvanny I., Vervliet T. (2021). The role of Bcl-2 proteins in modulating neuronal Ca2+ signaling in health and in Alzheimer’s disease. BBA-MOL. CELL. RES..

[21] Liu H., Wang S., Wang J., Guo X., Song Y., Fu K., Gao Z., Liu D., He W., Yang L.L. (2025). Energy Metabolism in Health and Diseases. Signal Transduct. Target. Ther..

[22] Limantoro J., de Liyis B.G., Sutedja J.C. (2023). Akt Signaling Pathway: A Potential Therapy for Alzheimer’s Disease through Glycogen Synthase Kinase 3 Beta Inhibition. Egypt. J. Neurol. Psychiatry Neurosurg..

[23] Tramutola A., Triplett J.C., Di Domenico F., Niedowicz D.M., Murphy M.P., Coccia R., Perluigi M., Allan Butterfield D. (2015). Alteration of MTOR Signaling Occurs Early in the Progression of Alzheimer Disease (AD): Analysis of Brain from Subjects with Pre-Clinical AD, Amnestic Mild Cognitive Impairment and Late-Stage AD. J. Neurochem..

[24] Davoody S., Asgari Taei A., Khodabakhsh P., Dargahi L. (2023). MTOR Signaling and Alzheimer’s Disease: What We Know and Where We Are?. CNS Neurosci. Ther..

[25] Deleyto-Seldas N., Efeyan A. (2021). The MTOR–Autophagy Axis and the Control of Metabolism. Front. Cell Dev. Biol..

[26] Xie P.L., Zheng M.Y., Han R., Chen W.X., Mao J.H. (2024). Pharmacological mTOR inhibitors in ameliorating Alzheimer’s disease: Current review and perspectives. Front. Pharmacol..

[27] Bas J., Nguyen T., Gillet G. (2021). Involvement of Bcl-XL in Neuronal Function and Development. Int. J. Mol. Sci..

[28] Li X., Wu Z., Si X., Wu G., Wang M. (2025). The role of mitochondrial dysfunction in the pathogenesis of Alzheimer’s disease and future strategies for targeted therapy. Eur. J. Med. Res..

[29] Liu S., Xu Y., Yao X., Cao H., Zhou H., Luo J., Gao H., Chen B., Chen H., Xie T. (2025). Perillaldehyde Ameliorates Sepsis-Associated Acute Kidney Injury via Inhibiting HSP90AA1-Mediated Ferroptosis and Pyroptosis: Molecular Structure and Protein Interaction of HSP90AA1. Int. J. Biol. Macromol..

[30] Singh M.K., Ranbhise J.S., Fu M., Ju S., Han S., Yun H.R., Choe W., Kim S.S., Kang I. (2025). Beyond Folding: Expanding the Functional Landscape of Hsp90 Chaperone Machinery in Health and Disease. Int. J. Mol. Sci..

[31] Soriano-Herrador C., Ubeda-Banon I., Villanueva-Anguita P., Saiz-Sanchez D., Astillero-Lopez V., Martinez-Marcos A., Flores-Cuadrado A. (2025). The Chaperones HSP90AA1, HSP90AB1 and BAG3 Are Specifically Distributed among Human Hippocampal Subfields during Different Alzheimer’s Disease Stages. Neurobiol. Dis..

[32] Maiti S., Picard D. (2022). Cytosolic Hsp90 Isoform-Specific Functions and Clinical Significance. Biomolecules.

[33] Di Lorenzo D., Bisi N., Kaffy J., Ramirez L.M., Zweckstetter M., Lequin O., Garfagnini I., Luo J., Hannappel Y., Ennen I. (2025). Synthetic Chaperone Based on Hsp90-Tau Interaction Inhibits Tau Aggregation and Rescues Physiological Tau-Microtubule Interaction. Nat. Commun..

[34] Gonzalez-Rodriguez M., Villar-Conde S., Astillero-Lopez V., Villanueva-Anguita P., Ubeda-Banon I., Flores-Cuadrado A., Martinez-Marcos A., Saiz-Sanchez D. (2021). Neurodegeneration and Astrogliosis in the Human CA1 Hippocampal Subfield Are Related to hsp90ab1 and bag3 in Alzheimer’s Disease. Int. J. Mol. Sci..

[35] Duan Y.W., Chen S.X., Li Q.Y., Zang Y. (2022). Neuroimmune Mechanisms Underlying Neuropathic Pain: The Potential Role of TNF-α-Necroptosis Pathway. Int. J. Mol. Sci..

[36] Habbas S., Santello M., Becker D., Stubbe H., Zappia G., Liaudet N., Klaus F.R., Kollias G., Fontana A., Pryce C.R. (2015). Neuroinflammatory TNFα Impairs Memory via Astrocyte Signaling. Cell.

[37] Guo J., Zhang H., Lin W., Lu L., Su J., Chen X. (2023). Signaling Pathways and Targeted Therapies for Psoriasis. Signal Transduct. Target. Ther..

[38] Chinnam M., Xu C., Lama R., Zhang X., Cedeno C.D., Wang Y., Stablewski A.B., Goodrich D.W., Wang X. (2022). MDM2 E3 Ligase Activity Is Essential for P53 Regulation and Cell Cycle Integrity. PLoS Genet..

[39] De Almeida J.F.M., Contestabile M., Tonazzini I., De Cesari C., Baroncelli L., Martini C., Daniele S. (2025). Dysfunction of the Autophagy System and MDM2–P53 Axis Leads to the Accumulation of Amyloidogenic Proteins in Angelman Syndrome Models. Int. J. Mol. Sci..

[40] Szybińska A., Leśniak W. (2017). P53 Dysfunction in Neurodegenerative Diseases—The Cause or Effect of Pathological Changes?. Aging Dis..

[41] Kommaddi R.P., Gowaikar R., Singh K., PA H., Ravindranath V. (2023). Down Regulation of Akt/MTOR Signaling Pathway Proteins in Hippocampus of Alzheimer’s Disease Mouse Model. Alzheimer’s Dement..

[42] De la Monte S.M., Tong M. (2024). Dysregulated mTOR networks in experimental sporadic Alzheimer’s disease. Front. Cell. Neurosci..

[43] Bhat I.A., Bhat A.M., Abdullah S.T., Bhat I.A., Bhat A.M., Abdullah S.T. (2025). Apoptosis-Mechanisms, Regulation in Pathology, and Therapeutic Potential. Cell Death Regulation in Pathology.

[44] Guo D., Liu Z., Zhou J., Ke C., Li D. (2024). Significance of Programmed Cell Death Pathways in Neurodegenerative Diseases. Int. J. Mol. Sci..

[45] Ziaka K., Van der Spuy J. (2022). The Role of Hsp90 in Retinal Proteostasis and Disease. Biomolecules.

[46] Zhang W., Xiao D., Mao Q., Xia H. (2023). Role of Neuroinflammation in Neurodegeneration Development. Signal Transduct. Target. Ther..

[47] Sharma A., Parekh B., Patil V., Renuka Jyothi S., Nayak P.P., Bethanney Janney J., Singh G., Al-Hasnaawei S. (2026). Astrocytes and Microglia in Alzheimer’s Disease: Friends, Foes, or Both?. Dev. Neurobiol..

[48] Too L.K., Hunt N., Simunovic M.P. (2021). The Role of Inflammation and Infection in Age-Related Neurodegenerative Diseases: Lessons From Bacterial Meningitis Applied to Alzheimer Disease and Age-Related Macular Degeneration. Front. Cell. Neurosci..

[49] Wen X., Zhang B., Wu B., Xiao H., Li Z., Li R., Xu X., Li T. (2022). Signaling Pathways in Obesity: Mechanisms and Therapeutic Interventions. Signal Transduct. Target. Ther..

[50] Li Y.Y., Qin Z.H., Sheng R. (2024). The Multiple Roles of Autophagy in Neural Function and Diseases. Neurosci. Bull..

[51] Zhang Z., Yang X., Song Y.Q., Tu J. (2021). Autophagy in Alzheimer’s Disease Pathogenesis: Therapeutic Potential and Future Perspectives. Ageing Res. Rev..

[52] Lior N., Chen D., Dan F., Ronit P.K. (2025). The Connection between Autophagy and Alzheimer’s Disease. Inflamm. Res..

[53] Cui J., Zhao C. (2025). Autophagy and Apoptosis in Alzheimer’s Disease-Associated Neurons. Discov. Neurosci..

[54] Gouda N.A., Zhakupova A., Abdelaal A.M., Ahmad F., Elkamhawy A. (2025). The Interplay Involving Oxidative Stress and Autophagy: Mechanisms, Implications, and Therapeutic Opportunities. Exp. Mol. Pathol..

[55] Zhang W., Xu C., Sun J., Shen H.M., Wang J., Yang C. (2022). Impairment of the Autophagy–Lysosomal Pathway in Alzheimer’s Diseases: Pathogenic Mechanisms and Therapeutic Potential. Acta Pharm. Sin. B.

[56] Zhou X.G., Qiu W.Q., Yu L., Pan R., Teng J.F., Sang Z.P., Law B.Y.K., Zhao Y., Zhang L., Yan L. (2022). Targeting Microglial Autophagic Degradation of the NLRP3 Inflammasome for Identification of Thonningianin A in Alzheimer’s Disease. Inflamm. Regen..

[57] Jiang G., Xie G., Li X., Xiong J. (2025). Cytoskeletal Proteins and Alzheimer’s Disease Pathogenesis: Focusing on the Interplay with Tau Pathology. Biomolecules.

[58] Zhang Z., Zhang M., Jin H., Lv S., Li Y., Li Y. (2025). Mitochondrial Quality Control and Cell Death. Int. J. Mol. Sci..

[59] Perez-Serna A.A., Guzman-Llorens D., Dos Santos R.S., Marroqui L. (2025). Bcl-2 and Bcl-XL in Diabetes: Contributions to Endocrine Pancreas Viability and Function. Biomedicines.

[60] Chernyuk D., Callens M., Polozova M., Gordeev A., Chigriai M., Rakovskaya A., Ilina A., Pchitskaya E., Van den Haute C., Vervliet T. (2023). Neuroprotective Properties of Anti-Apoptotic BCL-2 Proteins in 5xFAD Mouse Model of Alzheimer’s Disease. IBRO Neurosci. Rep..

[61] Beigl T.B., Paul A., Fellmeth T.P., Nguyen D., Barber L., Weller S., Schäfer B., Gillissen B.F., Aulitzky W.E., Kopp H. (2024). BCL-2 and BOK regulate apoptosis by interaction of their C-terminal transmembrane domains. EMBO Rep..

[62] Moll A., Ramirez L.M., Ninov M., Schwarz J., Urlaub H., Zweckstetter M. (2022). Hsp Multichaperone Complex Buffers Pathologically Modified Tau. Nat. Commun..

[63] Sola M., Rendon-Angel A., Rojo Martinez V., Sgrignani J., Magrin C., Piovesana E., Cavalli A., Paganetti P., Papin S. (2023). Tau Protein Binds to the P53 E3 Ubiquitin Ligase MDM2. Sci. Rep..

[64] Tewari D., Sah A.N., Bawari S., Nabavi S.F., Dehpour A.R., Shirooie S., Braidy N., Fiebich B.L., Vacca R.A., Nabavi S.M. (2021). Role of Nitric Oxide in Neurodegeneration: Function, Regulation, and Inhibition. Curr. Neuropharmacol..

[65] Opland C.K., Bryan M.R., Harris B., McGillion-Moore J., Tian X., Chen Y., Itano M.S., Diering G.H., Meeker R.B., Cohen T.J. (2023). Activity-Dependent Tau Cleavage by Caspase-3 Promotes Neuronal Dysfunction and Synaptotoxicity. iScience.

[66] Hossain M.S., Hussain M.H. (2025). Multi-Target Drug Design in Alzheimer’s Disease Treatment: Emerging Technologies, Advantages, Challenges, and Limitations. Pharmacol. Res. Perspect..

[67] PubChem. https://pubchem.ncbi.nlm.nih.gov/#query=Nevirapine.

[68] SwissTargetPrediction. http://www.swisstargetprediction.ch/.

[69] SEA Search Server. https://sea.bkslab.org/.

[70] GeneCards—Human Genes|Gene Database|Gene Search. https://www.genecards.org/.

[71] Venny 2.1.0. https://bioinfogp.cnb.csic.es/tools/venny/.

[72] Szklarczyk D., Kirsch R., Koutrouli M., Nastou K., Mehryary F., Hachilif R., Gable A.L., Fang T., Doncheva N.T., Pyysalo S. (2023). The STRING Database in 2023: Protein-Protein Association Networks and Functional Enrichment Analyses for Any Sequenced Genome of Interest. Nucleic Acids Res..

[73] Download Cytoscape. https://cytoscape.org/download.html.

[74] Ge S.X., Jung D., Jung D., Yao R. (2020). ShinyGO: A Graphical Gene-Set Enrichment Tool for Animals and Plants. Bioinformatics.

[75] Barretto A.J.B., Orda M.A., Tsai P.W., Tayo L.L. (2024). Analysis of Modular Hub Genes and Therapeutic Targets across Stages of Non-Small Cell Lung Cancer Transcriptome. Genes.

[76] Muley V.Y. (2025). Functional Insights Through Gene Ontology, Disease Ontology, and KEGG Pathway Enrichment. Methods Mol. Biol..

[77] Ni Y., Seffernick A.E., Onar-Thomas A., Pounds S.B. (2024). Computing Power and Sample Size for the False Discovery Rate in Multiple Applications. Genes.

[78] Enrichment Analysis. https://bamboo.genobank.org/enrichment.html.

[79] RCSB PDB: Homepage. https://www.rcsb.org/.

[80] Kannan D.C., Radhakrishnan M.S., Sambathkumar D.R., Dhanaraja M.D., Muvendhiran M.S., Dharnisha M.N.J. (2024). A Review on Step into the Future: Python Prescription (PyRx) Transforms Virtual Drug Discovery with AI-Driven Tools. Afr. J. Biomed. Res..

[81] Fitrianingsih S.P., Kurniati N.F., Fakih T.M., Adnyana I.K. (2025). Integrating Network Pharmacology, Molecular Docking, and Molecular Dynamics to Explore the Antidiabetic Mechanism of *Physalis angulata* L.. Pharmacia.

[82] Bhattacharya K., Chandra Nath B., Ahmed E., Khanal P., Chanu N.R., Deka S., Das D., Shrivastava A.K. (2024). Integration of Network Pharmacology, Molecular Docking, and Simulations to Evaluate Phytochemicals from Drymaria Cordata against Cervical Cancer. RSC Adv..

[83] Ugwu-Korie N., Quaye O., Wright E., Languon S., Agyapong O., Broni E., Gupta Y., Kempaiah P., Kwofie S.K. (2023). Structure-Based Identification of Natural-Product-Derived Compounds with Potential to Inhibit HIV-1 Entry. Molecules.

[84] Shana E. (2023). Protein-Ligand Interactions: Its Biological Process and Molecular Choreography for Drug Development to Cell Signaling. Enzym. Eng..

[85] BIOVIA Discovery Studio|Dassault Systèmes. https://www.3ds.com/products/biovia/discovery-studio.

