# Supplementary material for: Discontinued BACE1 Inhibitors in Phase II/III Clinical Trials and AM-6494 (Preclinical) Towards Alzheimer’s Disease Therapy: Repurposing Through Network Pharmacology and Molecular Docking Approach
Source: Pharmaceuticals (Basel). 2026 Jan 13;19(1):138. doi: 10.3390/ph19010138 (PMC12844986; doi:10.3390/ph19010138)
Supplement: Supplementary file 1 [file pharmaceuticals-19-00138-s001.zip › Figures S1-S5_Illustration of KEGG Pathways.pdf]

Discontinued BACE1 Inhibitors in Phase II/III Clinical Trials and AM-6494 (Preclinical) Towards Alzheimer's Disease Therapy: Repurposing Through Network Pharmacology and Molecular Docking Approach

Samuel Chima Ugbaja, Hezekiel Matambo Kumalo and Nceba Gqaleni

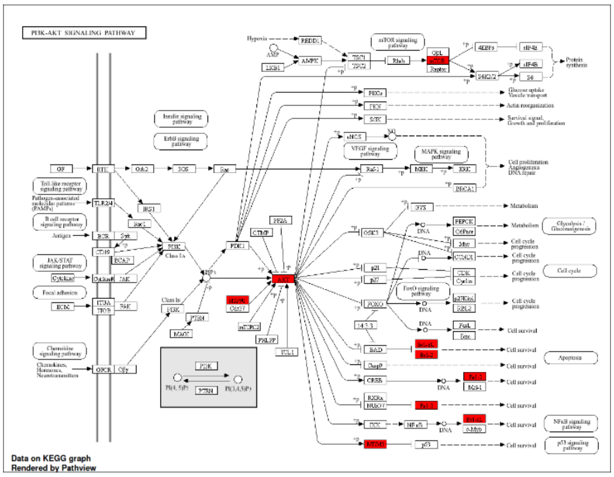

Figure S1. Illustration of P13K-AKT Signaling Pathway

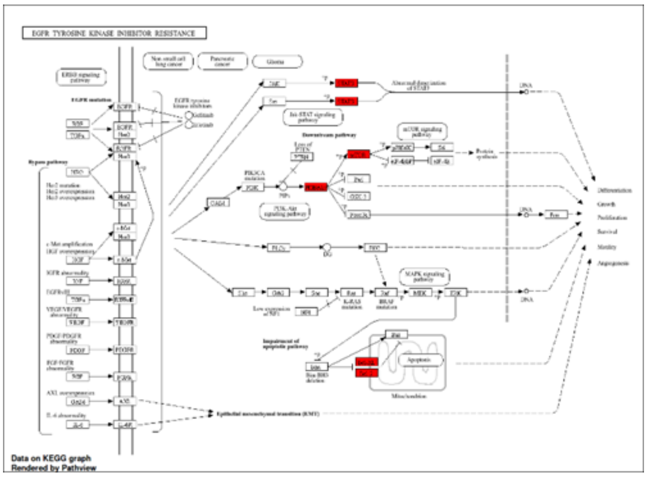

Figure S2. Illustration of EGFR tyrosine kinase inhibitor resistance

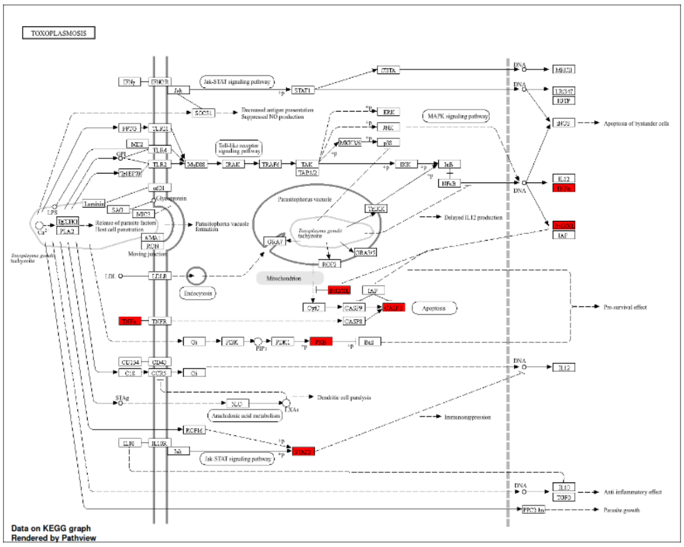

Figure S3. Illustration of Toxoplasmosis

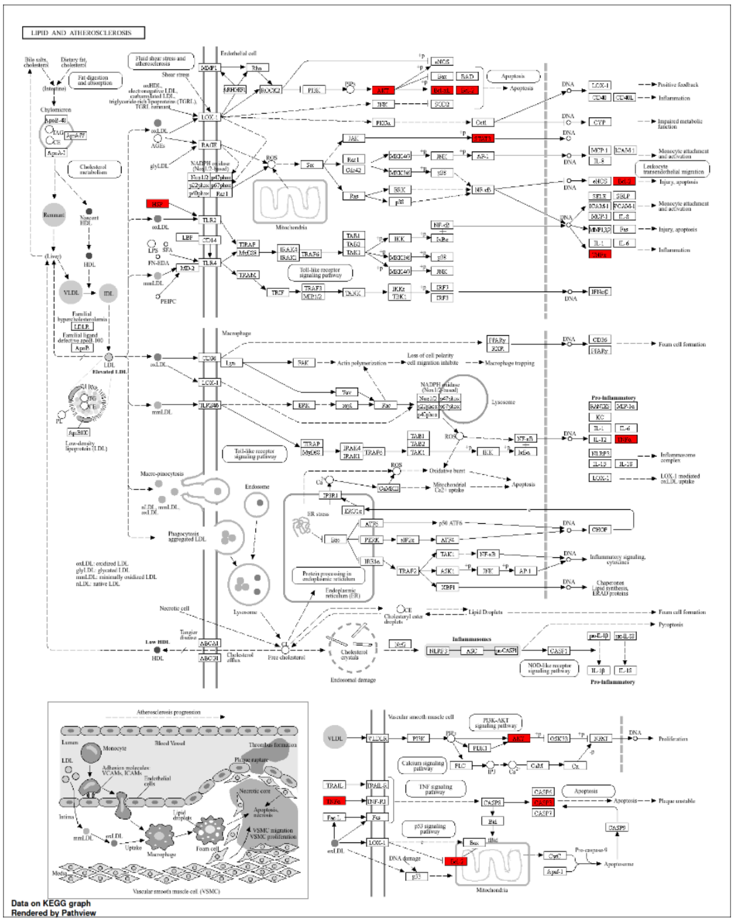

Figure S4. Illustration of Lipid and Atherosclerosis

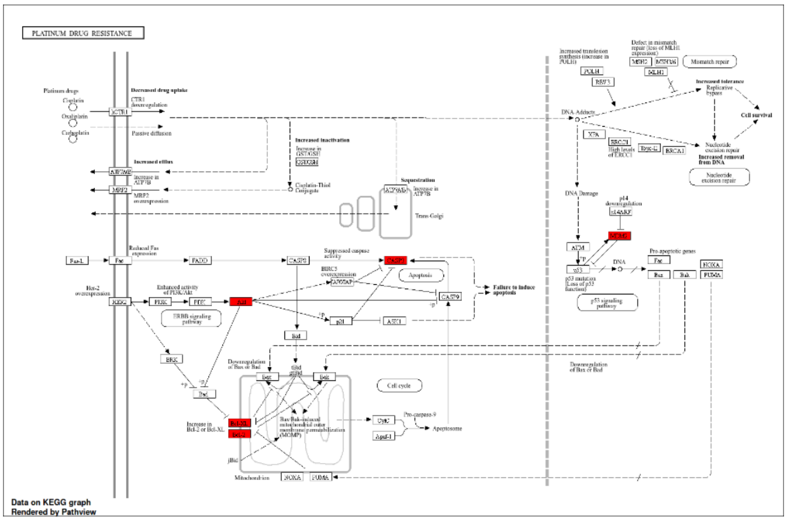

Figure S5. Illustration of Platinum drug resistance
