# Supplementary material for: Discontinued BACE1 Inhibitors in Phase II/III Clinical Trials and AM-6494 (Preclinical) Towards Alzheimer’s Disease Therapy: Repurposing Through Network Pharmacology and Molecular Docking Approach
Source: Pharmaceuticals (Basel). 2026 Jan 13;19(1):138. doi: 10.3390/ph19010138 (PMC12844986; doi:10.3390/ph19010138)
Supplement: Supplementary file 1 [file pharmaceuticals-19-00138-s001.zip › Figures S6-S11_2D Protein-Ligand Interaction Complex .pdf]

Discontinued BACE1 Inhibitors in Phase II/III Clinical Trials and AM-6494 (Preclinical) Towards Alzheimer's Disease Therapy: Repurposing Through Network Pharmacology and Molecular Docking Approach

Samuel Chima Ugbaja, Hezekiel Matambo Kumalo and Nceba Gqaleni

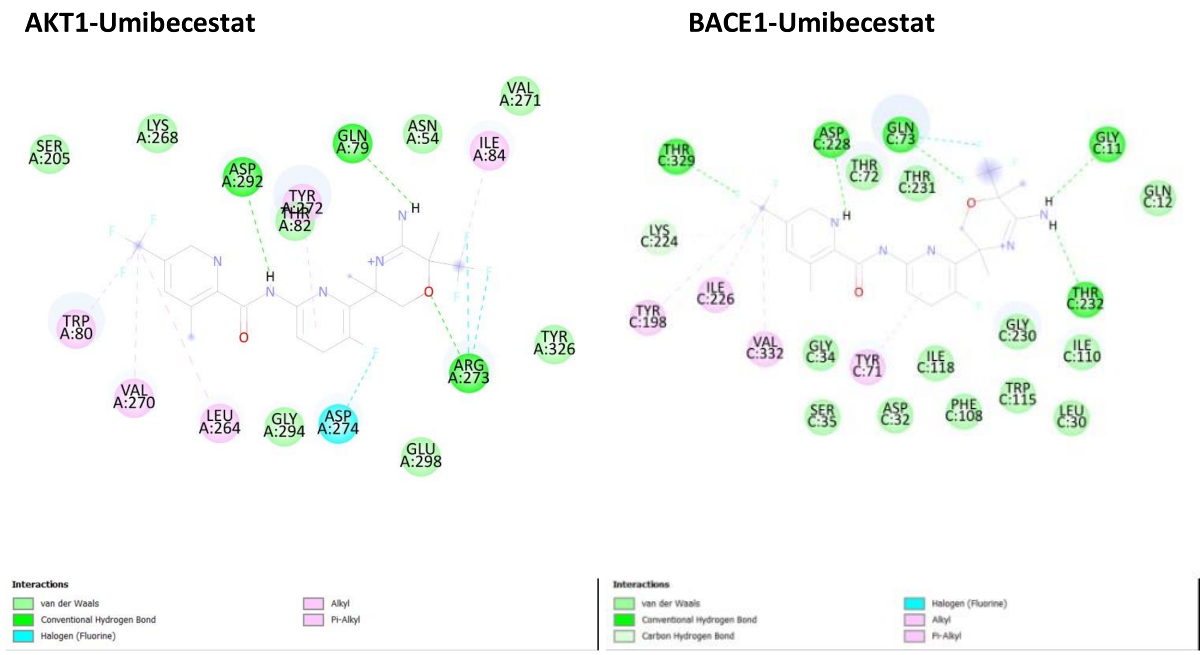

Figure S6. 2D representation of AKT1-Umibecestat and BACE1-Umibecestat complex

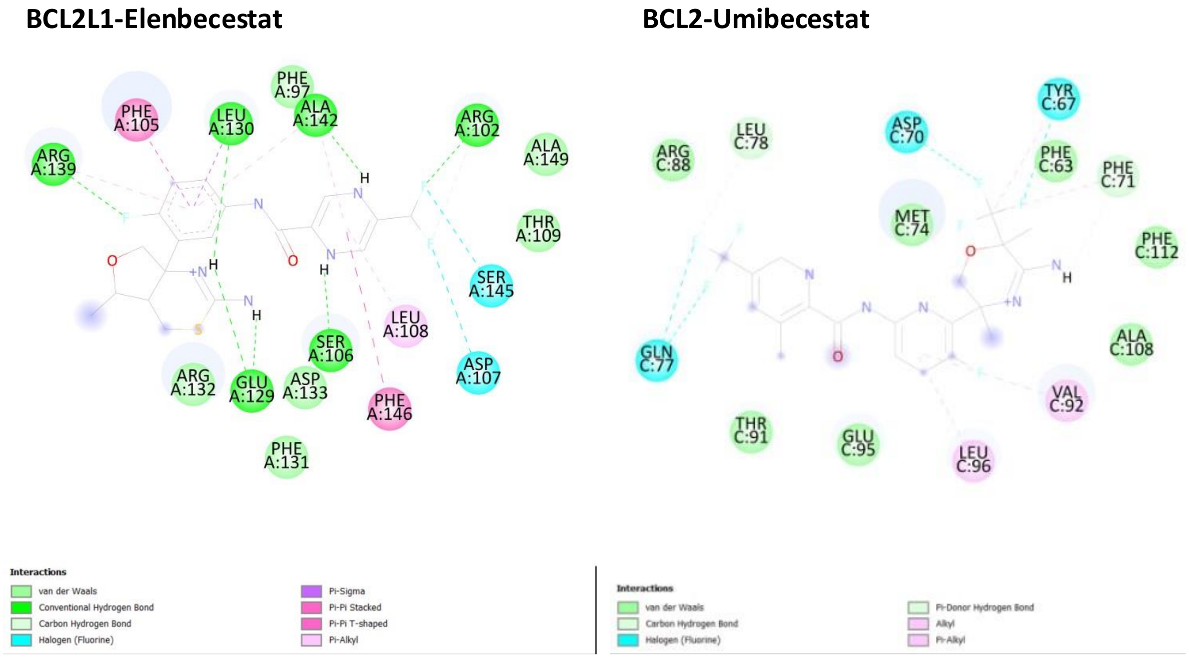

Figure S7. 2D representation of BCL2L1-Elenbecestat and BCL-Umibecestat complex

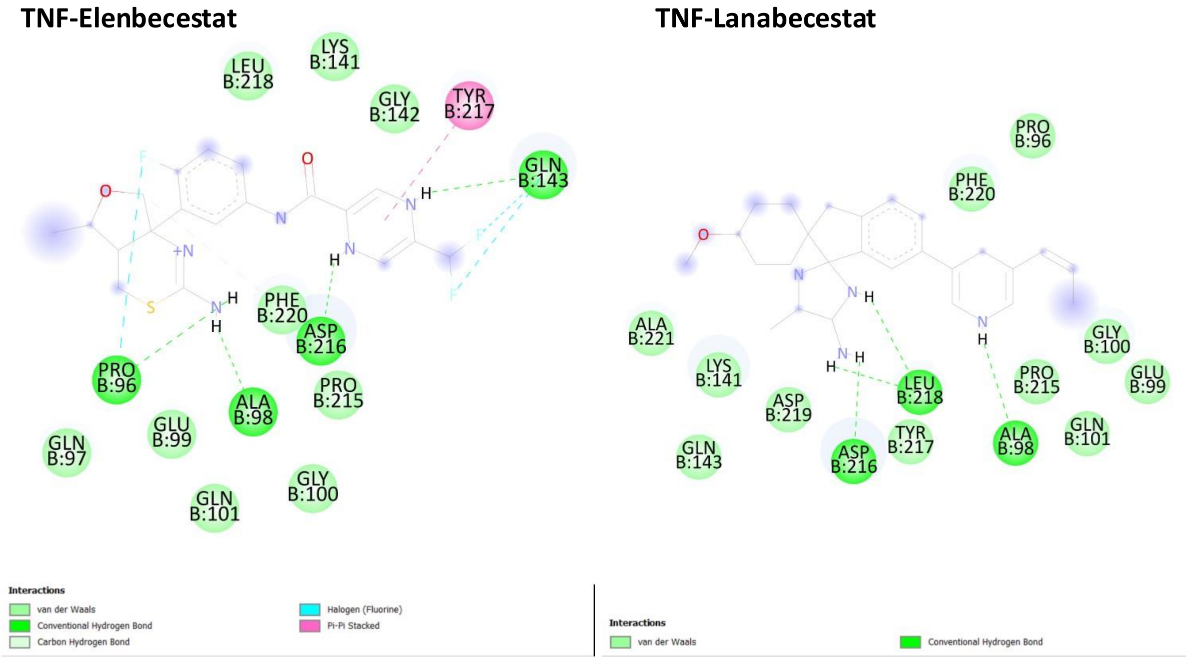

Figure S8. 2D representation of TNF-Elenbecestat and TNF-Lanabecestat complex

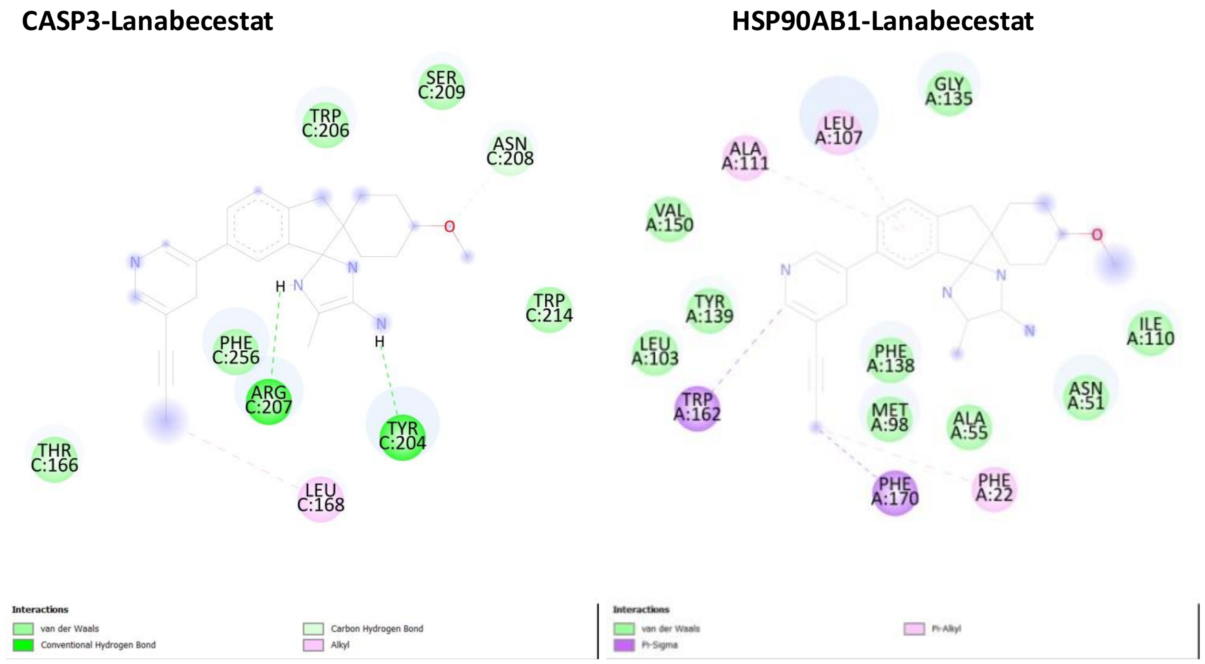

Figure S9. 2D representation of CASP3-Lanabecestat and HSP90AB1-Lanabecestat complex

HSP90AA1-Umibecestat **MDM2-Umibecestat**

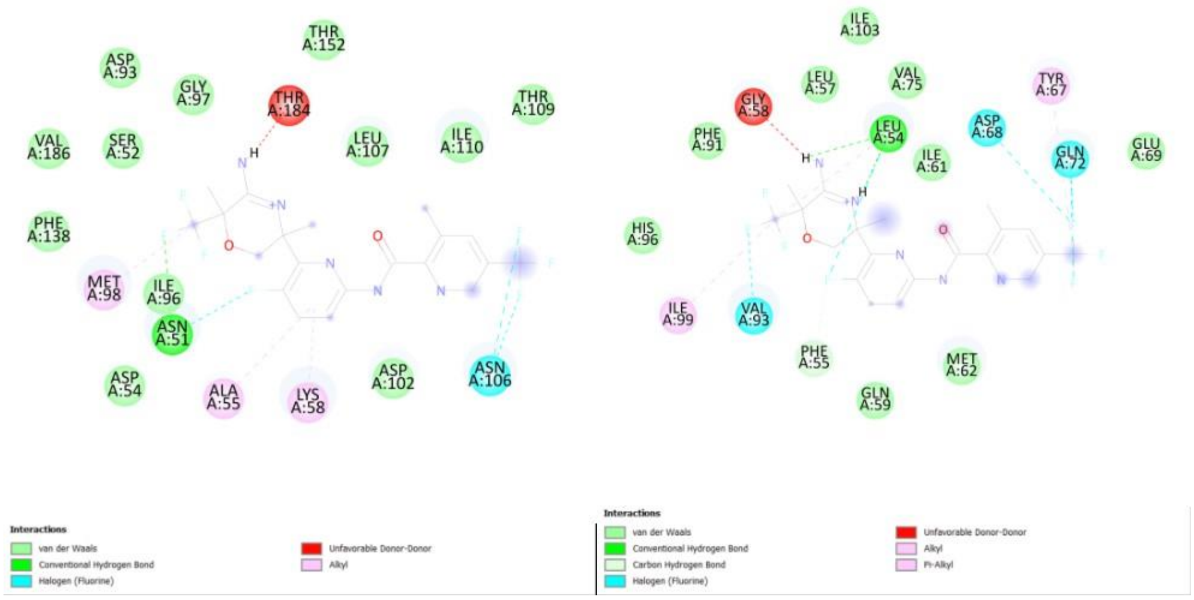

Figure S10. 2D representation of HSP90AA1-Umibecestat and MDM2-Umibecestat complex

MTOR-Umibecestat **STAT3-Umibecestat**

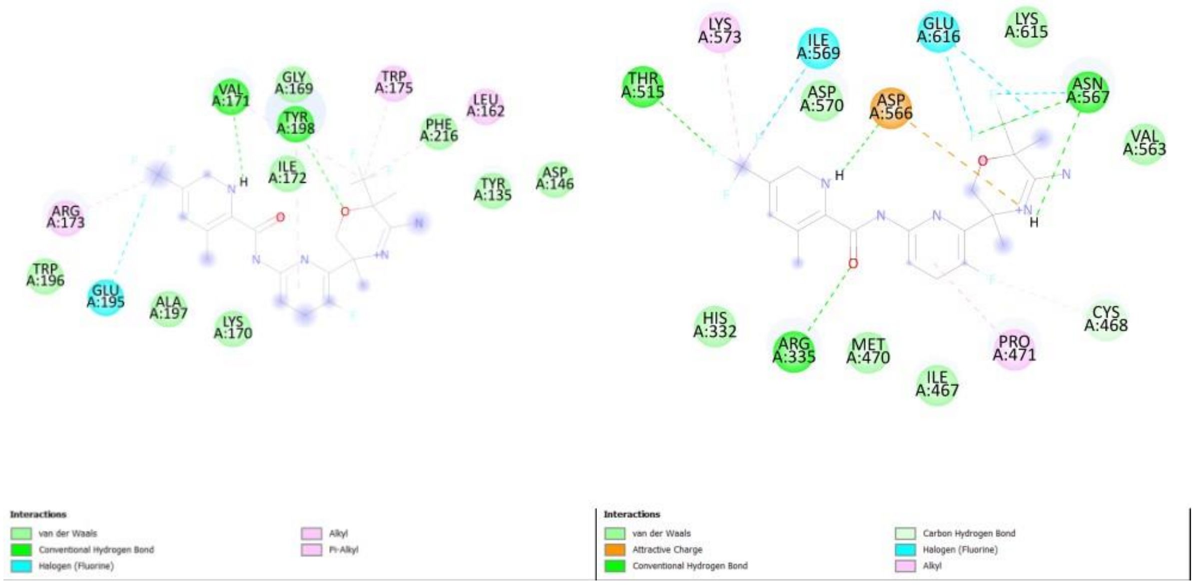

Figure S11. 2D representation of MTOR-Umibecestat and STAT3-Umibecestat complex
